# Supplementary material for: Genetic Implication of Prenatal GABAergic and Cholinergic Neuron Development in Susceptibility to Schizophrenia
Source: Schizophr Bull. 2024 Jun 13;50(5):1171–84. doi: 10.1093/schbul/sbae083 (PMC11349020; doi:10.1093/schbul/sbae083)
Supplement: sbae083_suppl_Supplementary_Figures [file sbae083_suppl_supplementary_figures.docx]

**Supplementary Figures**


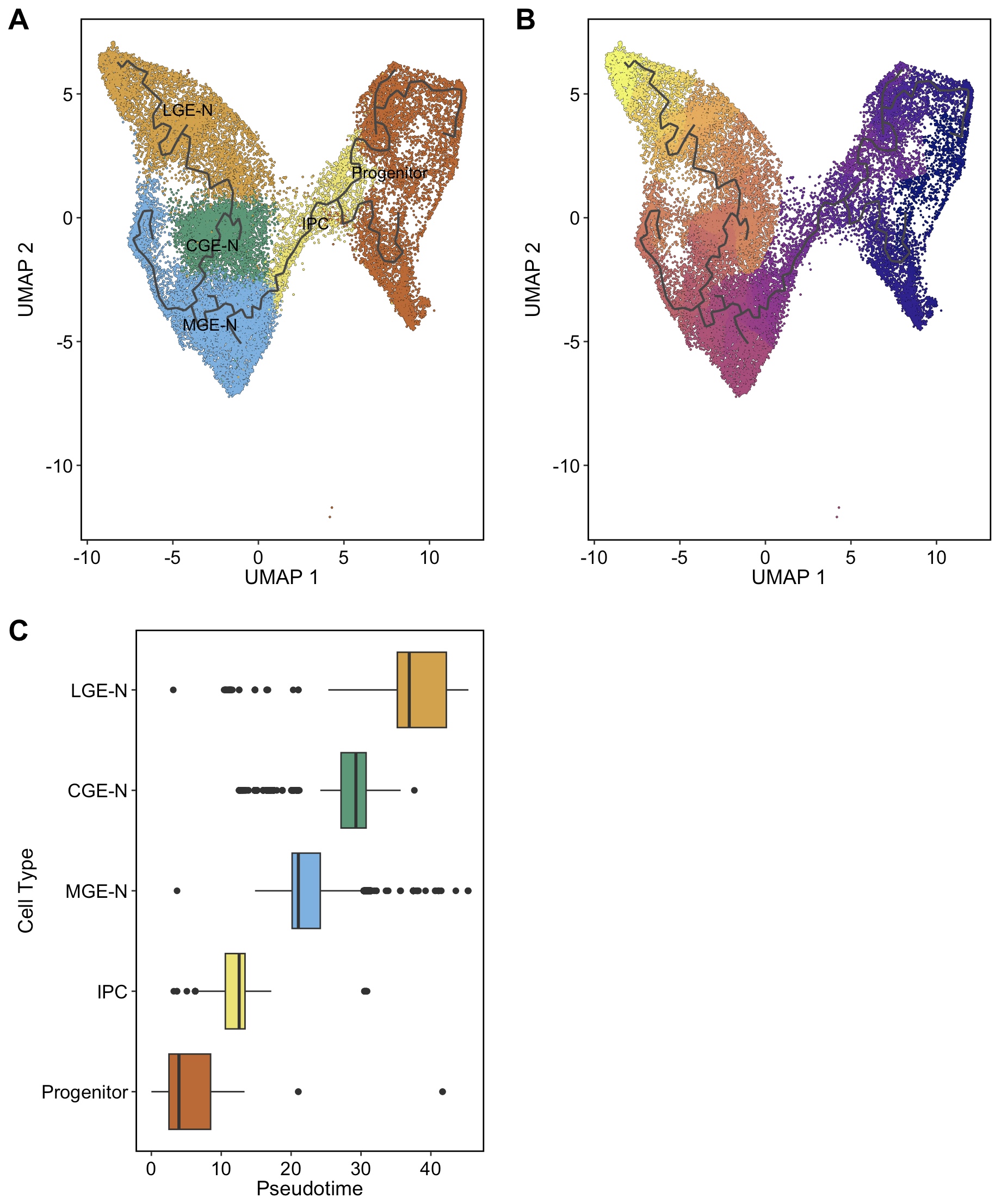


**Figure S1 – Developmental trajectories of level 1 cell populations of the human ganglionic eminences.** Trajectory inference analysis was performed using Monocle 3 (Cao et al, 2019). A) The Seurat object was converted into a cell_data_set object using the SeuratWrappers function, with level 1 cluster labels and UMAP co-ordinates retained. Microglia were removed due to their distinct origin. B) A single, multi-branched, trajectory graph progressing from progenitor cells, thorough intermediate progenitor cells, with branches terminating in post-mitotic neuronal populations of the medial, lateral and caudal portions of the ganglionic eminences was established. C) Pseudotime analyses predict that the bulk of medial ganglionic eminence neurons emerge before that of the caudal and lateral portions. CGE-N = developing neurons from the CGE; LGE-N = developing neurons from the LGE; MGE-N = developing neurons from the MGE; IPC = intermediate progenitor cells.

**Reference:**

Cao J, Spielmann M, Qiu X, et al. The single-cell transcriptional landscape of mammalian organogenesis. *Nature* 2019;566:496-502.


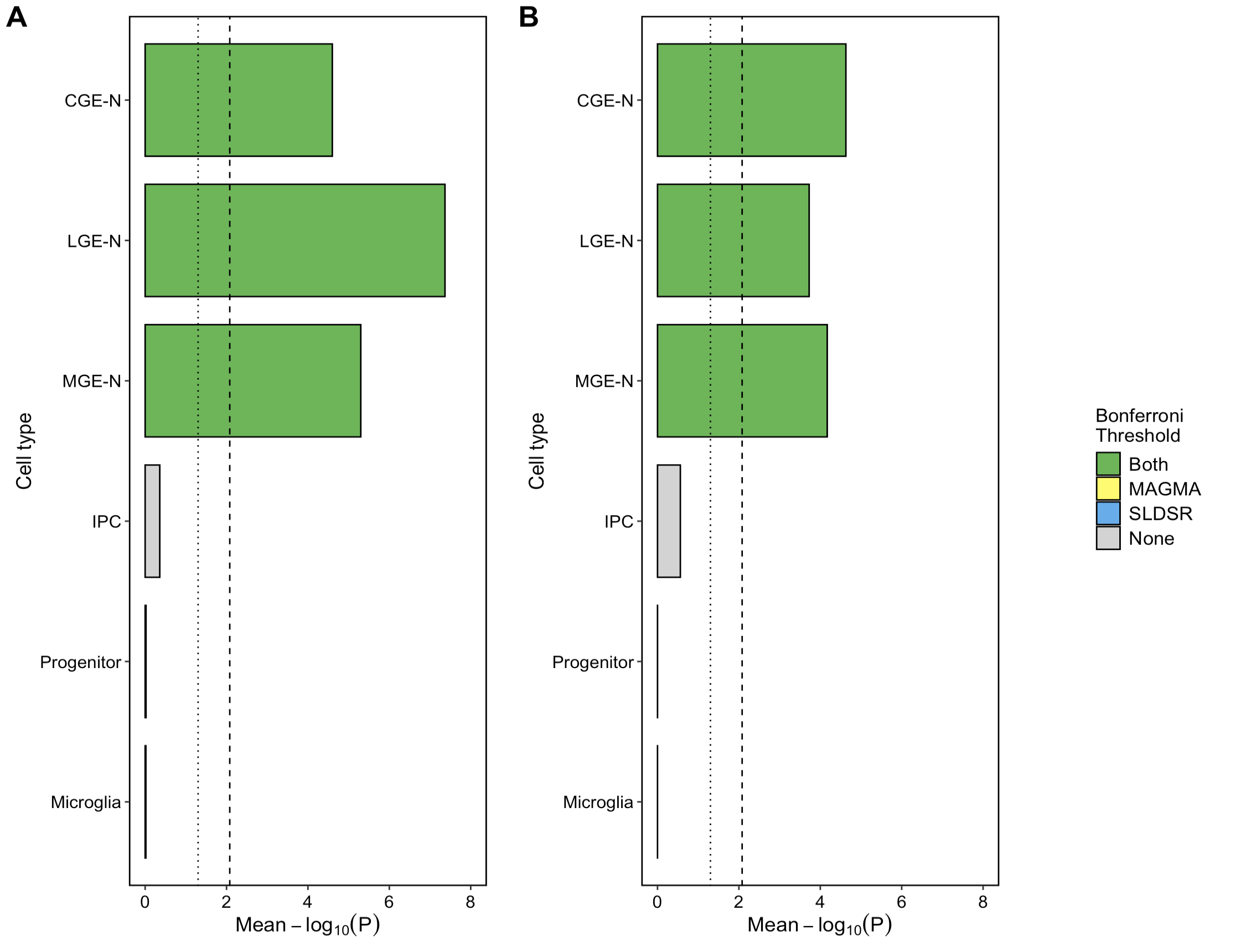


**Figure S2. Mean of the MAGMA (de Leeuw et al, 2015) and SLDSR (Finucane et al, 2018) -log₁₀ *P*-values for enrichment of schizophrenia common variant liability in genes in the top expression specificity decile of broad level 1 cell types of the ganglionic eminences after (A) downsampling the number of cells and (B) standardizing the number of genes included for each cell population.** A) Mean -log_10_ *P*-values when all level 1 cell types have been down-sampled to match the cell population with the lowest number of cells (microglia, with 242 cells). B) Mean -log_10_ *P*-values when the 1000 genes with the highest specificity scores for each cell population are analysed. The dotted vertical line indicates nominal (*P* < 0.05) significance and the dashed vertical line indicates the Bonferroni-corrected *P*-value threshold for 6 tested cell populations at level 1 (*P* < 0.0083). Bars are color-coded according to whether the enrichments are significant at the Bonferroni-corrected threshold for both MAGMA and SLDSR (green), MAGMA only (yellow), SLDSR only (blue) or neither test (grey). CGE-N = developing neurons from the CGE; LGE-N = developing neurons from the LGE; MGE-N = developing neurons from the MGE; IPC = intermediate progenitor cells.

**References:**

de Leeuw CA, Mooij JM, Heskes T, Posthuma D. MAGMA: generalized gene-set analysis of GWAS data. *PLoS Comput Biol* 2015;11:e1004219.

Finucane HK, Reshef YA, Anttila V, et al. Heritability enrichment of specifically expressed genes identifies disease-relevant tissues and cell types. *Nat Genet* 2018;50:621-629.


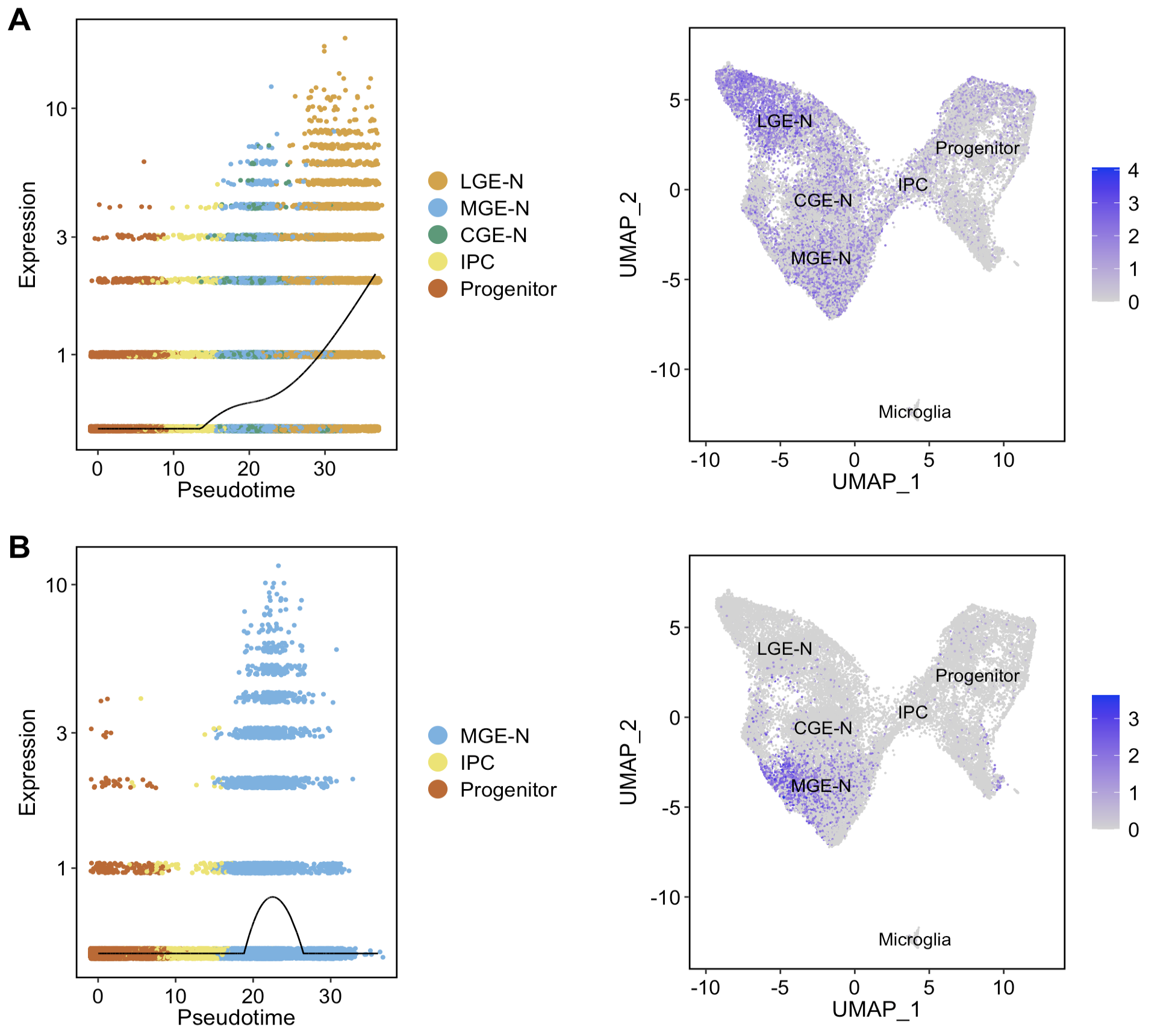


**Figure S3. Exemplar genes prioritized by Trubetskoy et al (2022) as candidates for schizophrenia susceptibility that display differential gene expression across developmental trajectories in the ganglionic eminences.** A) Expression of *BCL11B* in cells of the ganglionic eminences across pseudotime (left panel) and in defined level 1 clusters (right panel). B) Expression of *NXPH1* in cells of the ganglionic eminences across pseudotime (left panel) and in defined level 1 clusters (right panel). CGE-N = developing neurons from the CGE; LGE-N = developing neurons from the LGE; MGE-N = developing neurons from the MGE; IPC = intermediate progenitor cells.

**Reference:**

Trubetskoy V, Pardiñas AF, Qi T, et al. Mapping genomic loci implicates genes and synaptic biology in schizophrenia. *Nature* 2022;604:502-508.


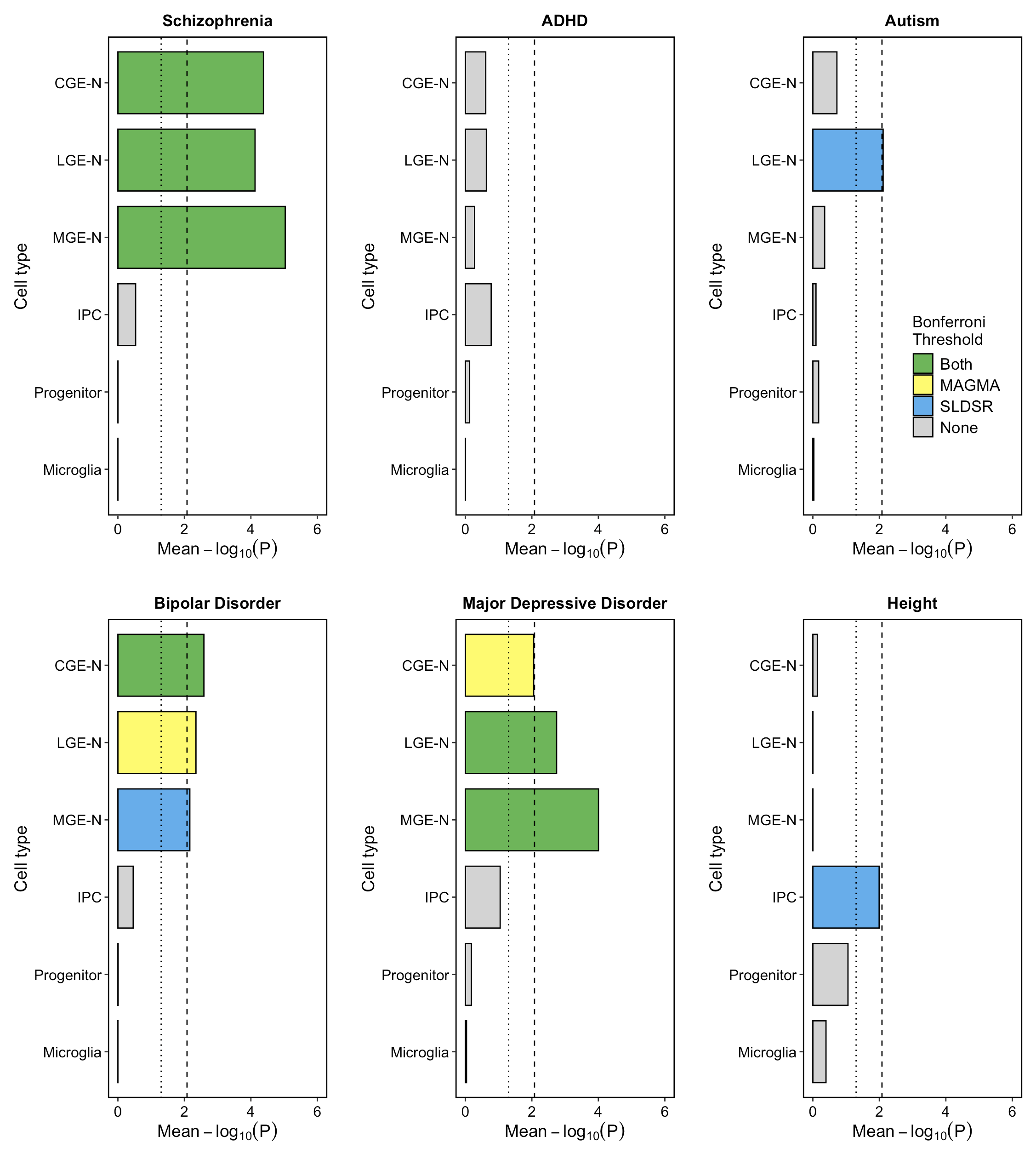


**Figure S4. Mean of the MAGMA (de Leeuw et al, 2015) and SLDSR (Finucane et al, 2018) -log₁₀ *P*-values for enrichment of genetic associations for schizophrenia and comparison phenotypes in genes with high expression specificity for level 1 cell types of the ganglionic eminences.** The dotted vertical line indicates nominal (*P* < 0.05) significance and the dashed vertical line indicates the Bonferroni-corrected *P*-value threshold for 6 tested cell populations for each phenotype (*P* < 0.0083). Bars are color-coded according to whether the enrichments are significant at the Bonferroni-corrected threshold for both MAGMA and SLDSR (green), MAGMA only (yellow), SLDSR only (blue) or neither test (grey). CGE-N = developing neurons from the CGE; LGE-N = developing neurons from the LGE; MGE-N = developing neurons from the MGE; IPC = intermediate progenitor cells; ADHD = attention deficit hyperactivity disorder.

**References:**

de Leeuw CA, Mooij JM, Heskes T, Posthuma D. MAGMA: generalized gene-set analysis of GWAS data. *PLoS Comput Biol* 2015;11:e1004219.

Finucane HK, Reshef YA, Anttila V, et al. Heritability enrichment of specifically expressed genes identifies disease-relevant tissues and cell types. *Nat Genet* 2018;50:621-629.


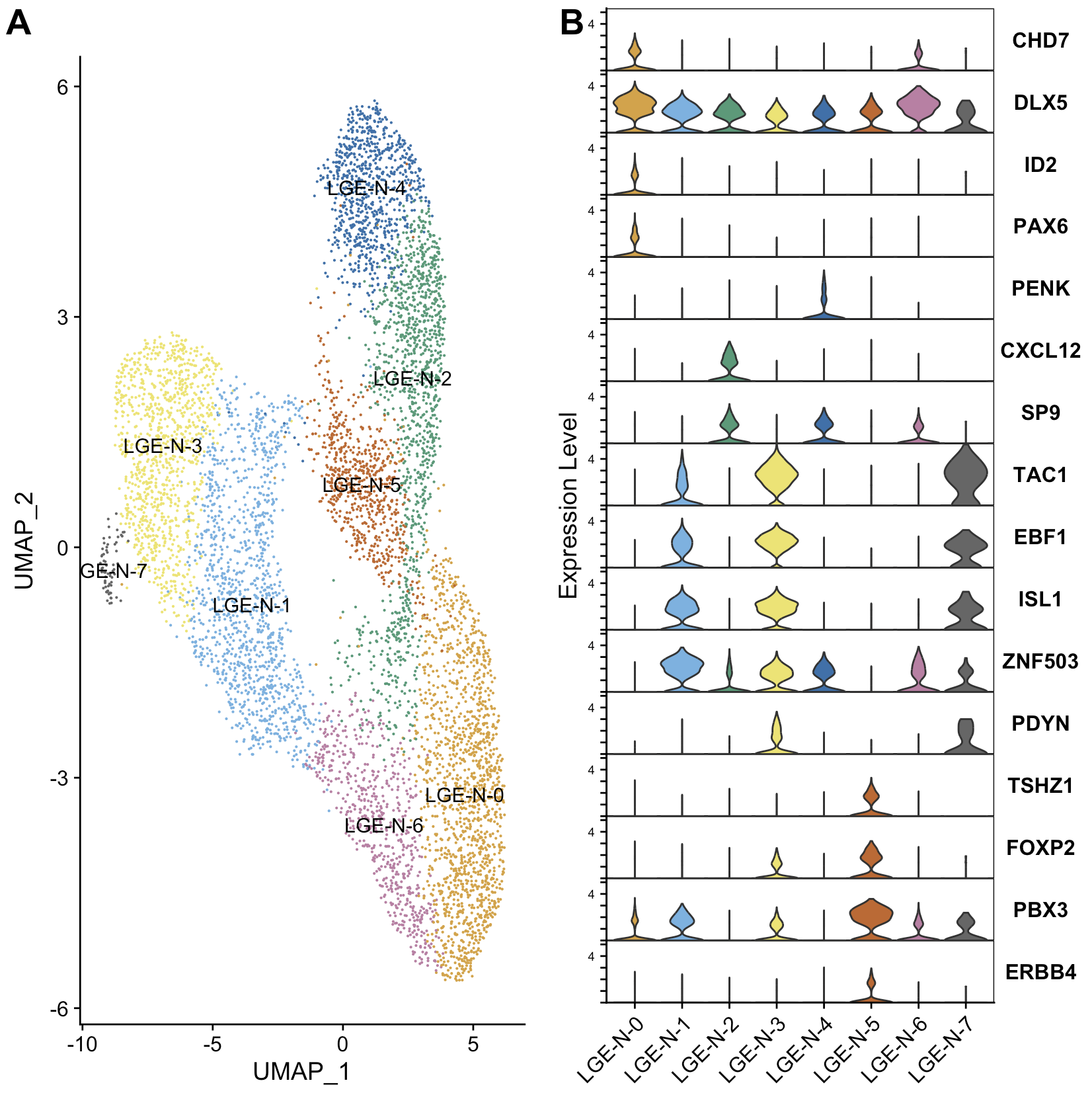


**Figure S5:** **Subclustering of single-cell RNA-Seq data from developing neurons of the lateral ganglionic eminence.** Cells were clustered according to gene expression profile using Seurat 4.3.0 (Hao et al, 2021) and visualized in two-dimensional space using UMAP. A) Clusters for level 2 lateral ganglionic eminence neuron (LGE-N) populations. B) Violin plots showing cell marker gene expression across the 8 level 2 lateral ganglionic eminence neuron (LGE-N) subpopulations.

**Reference:**

Hao Y, Hao S, Andersen-Nissen E, et al. Integrated analysis of multimodal single-cell data. *Cell* 2021;184:3573-3587.e29.


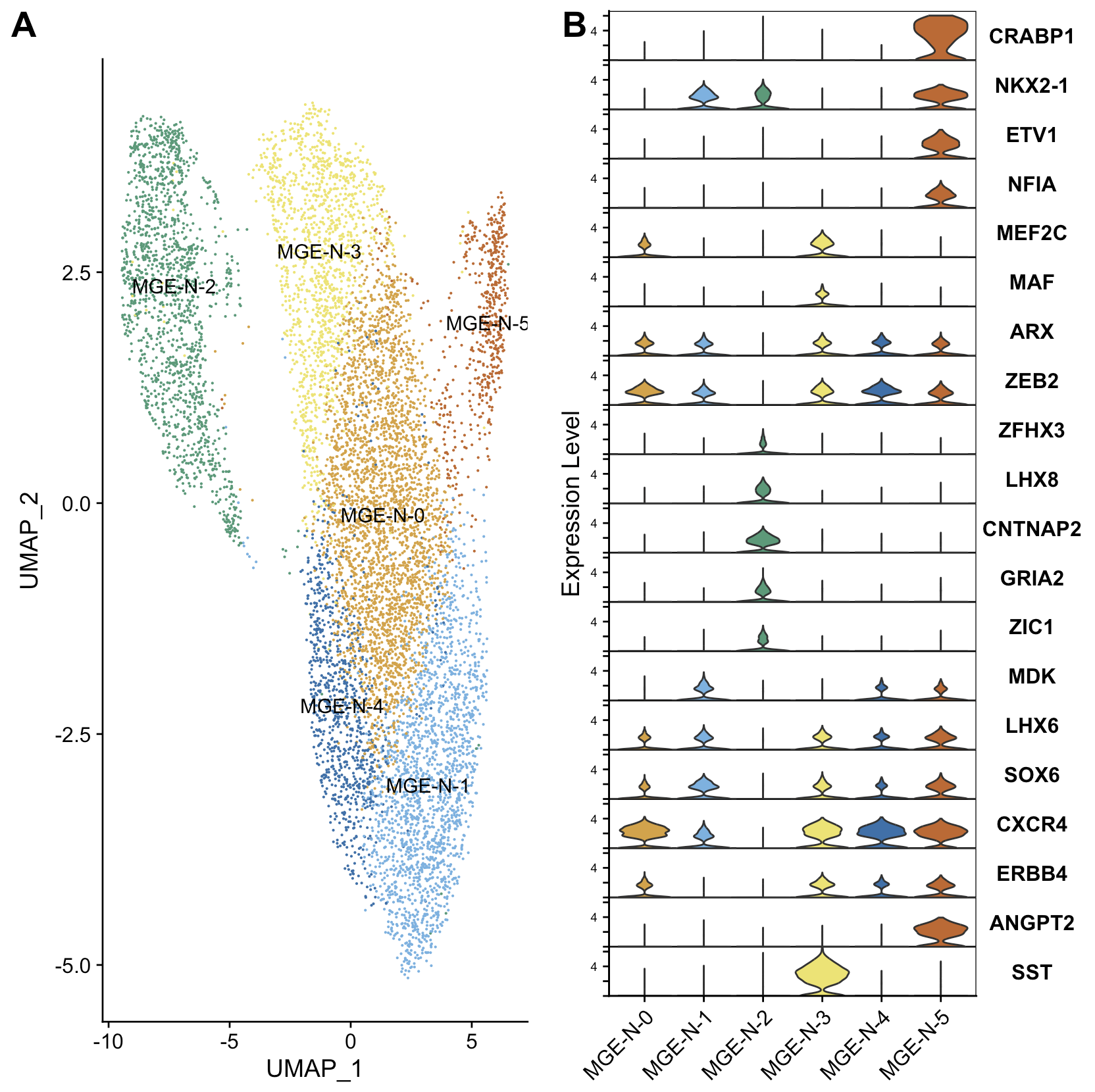


**Figure S6:** **Subclustering of single-cell RNA-Seq data from developing neurons of the medial ganglionic eminence.** Cells were clustered according to gene expression profile using Seurat 4.3.0 (Hao et al, 2021) and visualized in two-dimensional space using UMAP. A) Clusters for level 2 medial ganglionic eminence neuron (MGE-N) populations. B) Violin plots showing cell marker gene expression across the 6 level 2 medial ganglionic eminence neuron (MGE-N) subpopulations.

**Reference:**

Hao Y, Hao S, Andersen-Nissen E, et al. Integrated analysis of multimodal single-cell data. *Cell* 2021;184:3573-3587.e29.


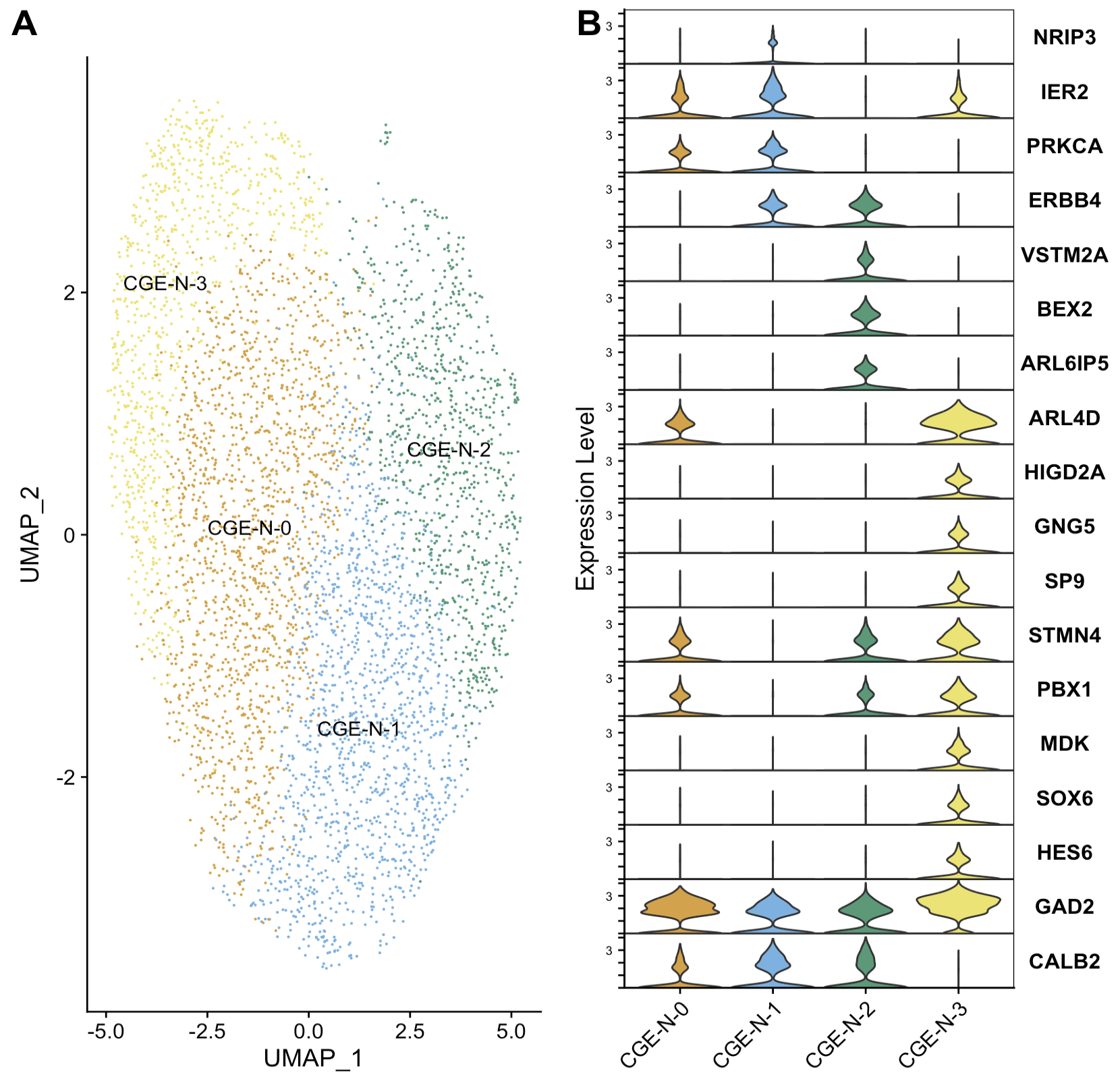


**Figure S7:** **Subclustering of single-cell RNA-Seq data from developing neurons of the caudal ganglionic eminence.** Cells were clustered according to gene expression profile using Seurat 4.3.0 (Hao et al, 2021) and visualized in two-dimensional space using UMAP. A) Clusters for level 2 caudal ganglionic eminence neuron (CGE-N) populations. B) Violin plots showing cell marker gene expression across the 4 level 2 caudal ganglionic eminence neuron (CGE-N) subpopulations.

**Reference:**

Hao Y, Hao S, Andersen-Nissen E, et al. Integrated analysis of multimodal single-cell data. *Cell* 2021;184:3573-3587.e29.


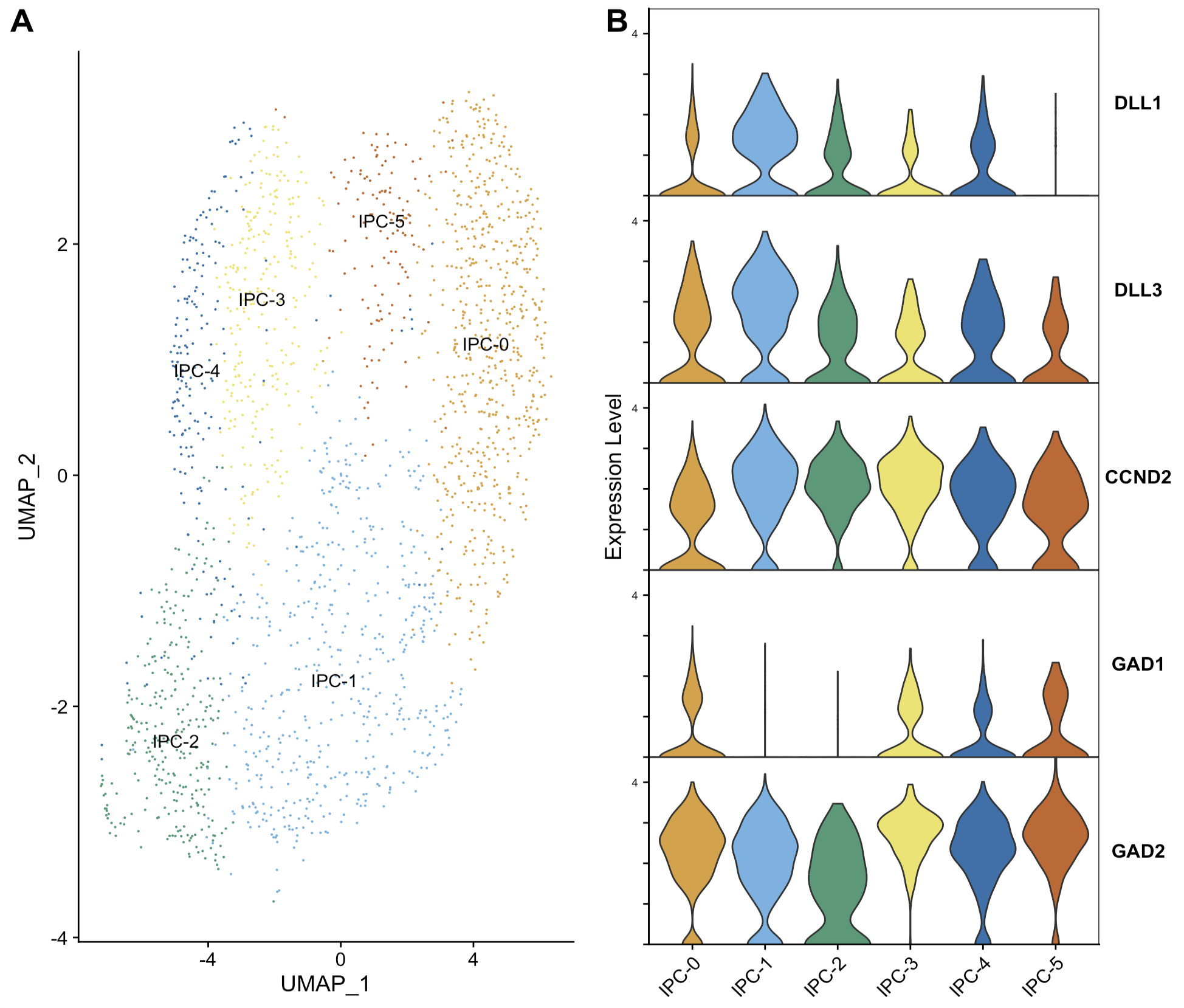


**Figure S8:** **Subclustering of single-cell RNA-Seq data from intermediate progenitor cells (IPC) of the ganglionic eminences.** Cells were clustered according to gene expression profile using Seurat 4.3.0 (Hao et al, 2021) and visualized in two-dimensional space using UMAP. A) Clusters for level 2 ganglionic eminence IPC populations. B) Violin plots showing cell marker gene expression across the 6 level 2 ganglionic eminence IPC subpopulations.

**Reference:**

Hao Y, Hao S, Andersen-Nissen E, et al. Integrated analysis of multimodal single-cell data. *Cell* 2021;184:3573-3587.e29.

**
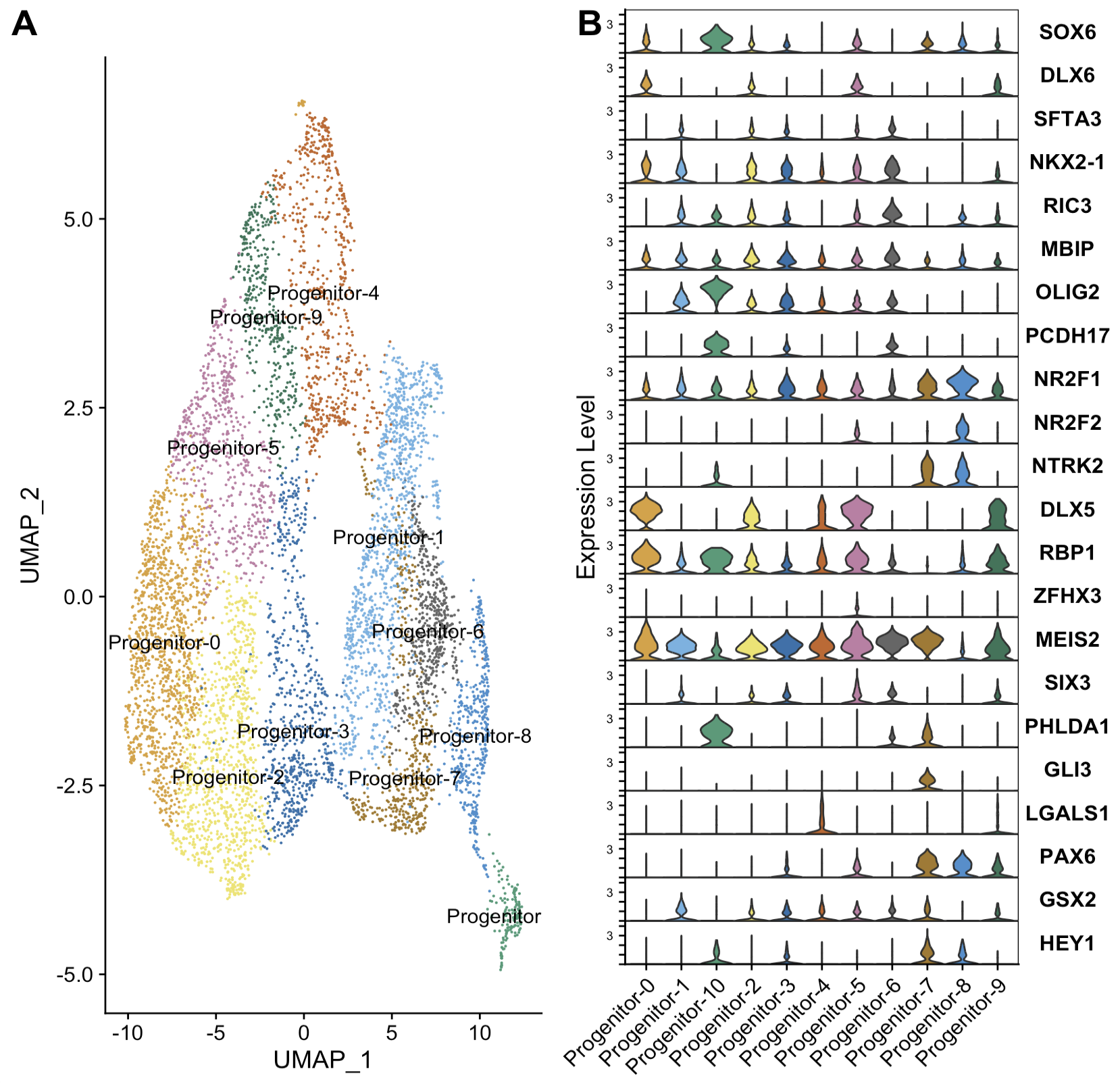
**

**Figure S9:** **Subclustering of single-cell RNA-Seq data from progenitor cells of the ganglionic eminences.** Cells were clustered according to gene expression profile using Seurat 4.3.0 (Hao et al, 2021) and visualized in two-dimensional space using UMAP. A) Clusters for level 2 ganglionic eminence progenitor cell populations. B) Violin plots showing cell marker gene expression across the 11 level 2 ganglionic eminence progenitor cell subpopulations.

**Reference:**

Hao Y, Hao S, Andersen-Nissen E, et al. Integrated analysis of multimodal single-cell data. *Cell* 2021;184:3573-3587.e29.


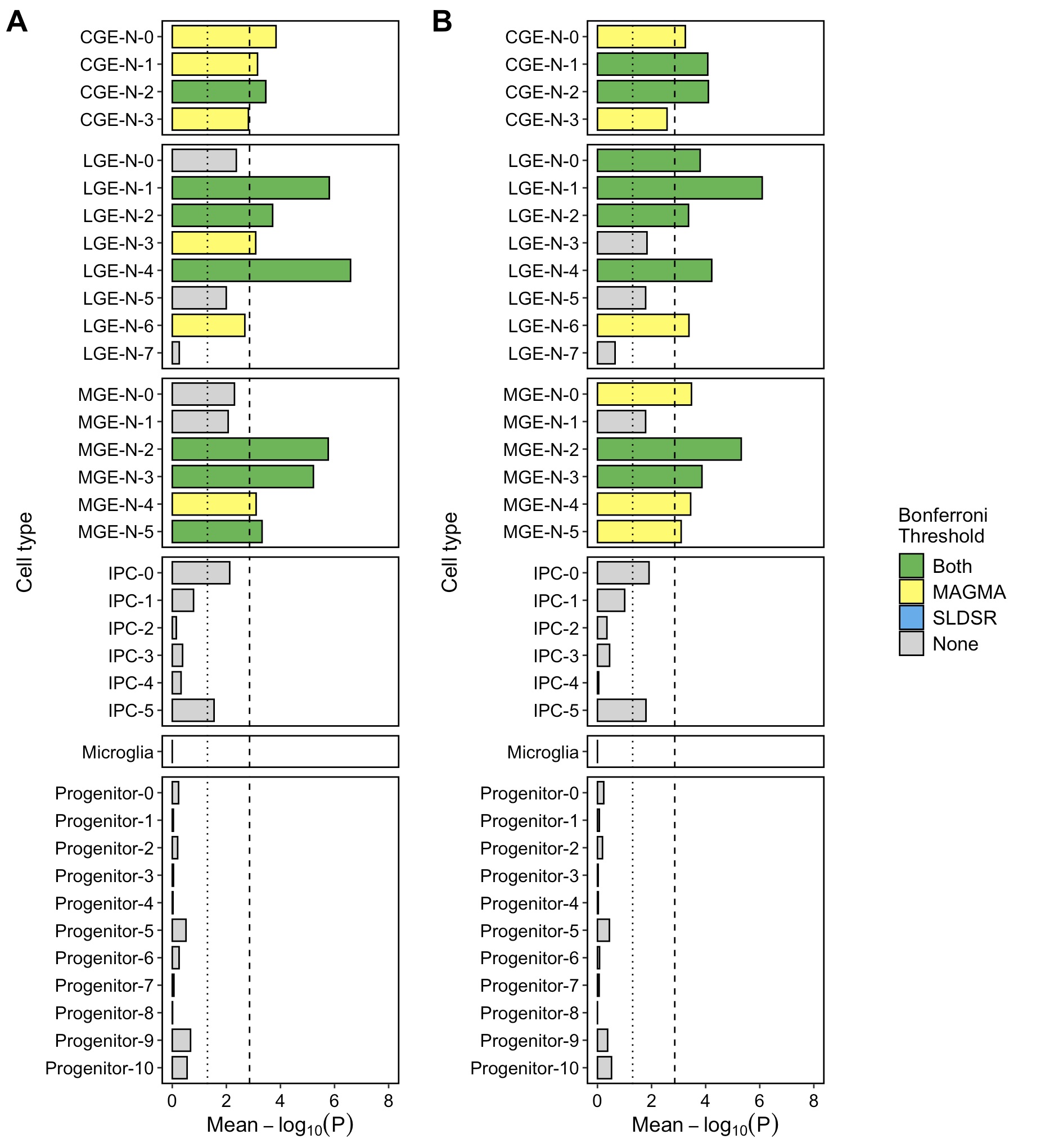


**Figure S10: Mean of the MAGMA (de Leeuw et al, 2015) and SLDSR (Finucane et al, 2018) -log₁₀ *P*-values for enrichment of schizophrenia common variant liability in genes in the top expression specificity decile of level 2 cell types of the ganglionic eminences after (A) downsampling the number of cells and (B) standardizing the number of genes included for each cell population.** A) Mean -log_10_ *P*-values when all level 1 cell types have been down-sampled to match the cell population with the lowest number of cells (microglia, with 242 cells). B) Mean -log_10_ *P*-values when the 1000 genes with the highest specificity scores for each cell population are analysed. The dotted vertical line indicates nominal (*P* < 0.05) significance and the dashed vertical line indicates the Bonferroni-corrected *P*-value threshold for 36 tested cell populations (*P* < 0.0014). Bars are color-coded according to whether the enrichments are significant at the Bonferroni-corrected threshold for both MAGMA and SLDSR (green), MAGMA only (yellow), SLDSR only (blue) or neither test (grey). CGE-N = developing neurons from the CGE; LGE-N = developing neurons from the LGE; MGE-N = developing neurons from the MGE; IPC = intermediate progenitor cells.

**References:**

de Leeuw CA, Mooij JM, Heskes T, Posthuma D. MAGMA: generalized gene-set analysis of GWAS data. *PLoS Comput Biol* 2015;11:e1004219.

Finucane HK, Reshef YA, Anttila V, et al. Heritability enrichment of specifically expressed genes identifies disease-relevant tissues and cell types. *Nat Genet* 2018;50:621-629.


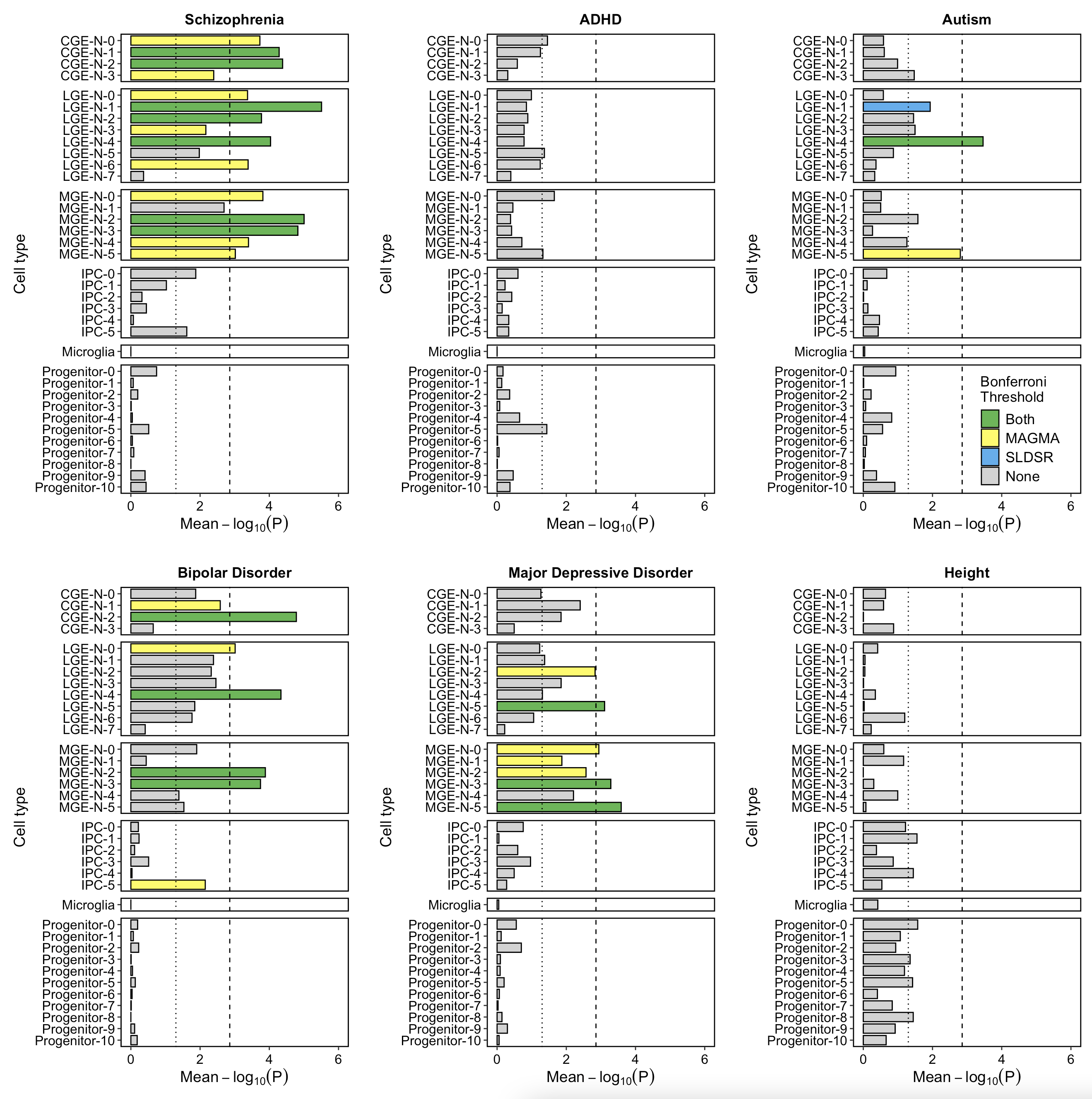


**Figure S11. Mean of the MAGMA (de Leeuw et al, 2015) and SLDSR (Finucane et al, 2018) -log₁₀ *P*-values for enrichment of genetic associations for schizophrenia and comparison phenotypes in genes with high expression specificity for level 2 cell types of the ganglionic eminences.** The dotted vertical line indicates nominal (*P* < 0.05) significance and the dashed vertical line indicates the Bonferroni-corrected *P*-value threshold for 36 tested cell populations for each phenotype (*P* < 0.0014). Bars are color-coded according to whether the enrichments are significant at the Bonferroni-corrected threshold for both MAGMA and SLDSR (green), MAGMA only (yellow), SLDSR only (blue) or neither test (grey). CGE-N = developing neurons from the CGE; LGE-N = developing neurons from the LGE; MGE-N = developing neurons from the MGE; IPC = intermediate progenitor cells; ADHD = attention deficit hyperactivity disorder.

**References:**

de Leeuw CA, Mooij JM, Heskes T, Posthuma D. MAGMA: generalized gene-set analysis of GWAS data. *PLoS Comput Biol* 2015;11:e1004219.

Finucane HK, Reshef YA, Anttila V, et al. Heritability enrichment of specifically expressed genes identifies disease-relevant tissues and cell types. *Nat Genet* 2018;50:621-629.


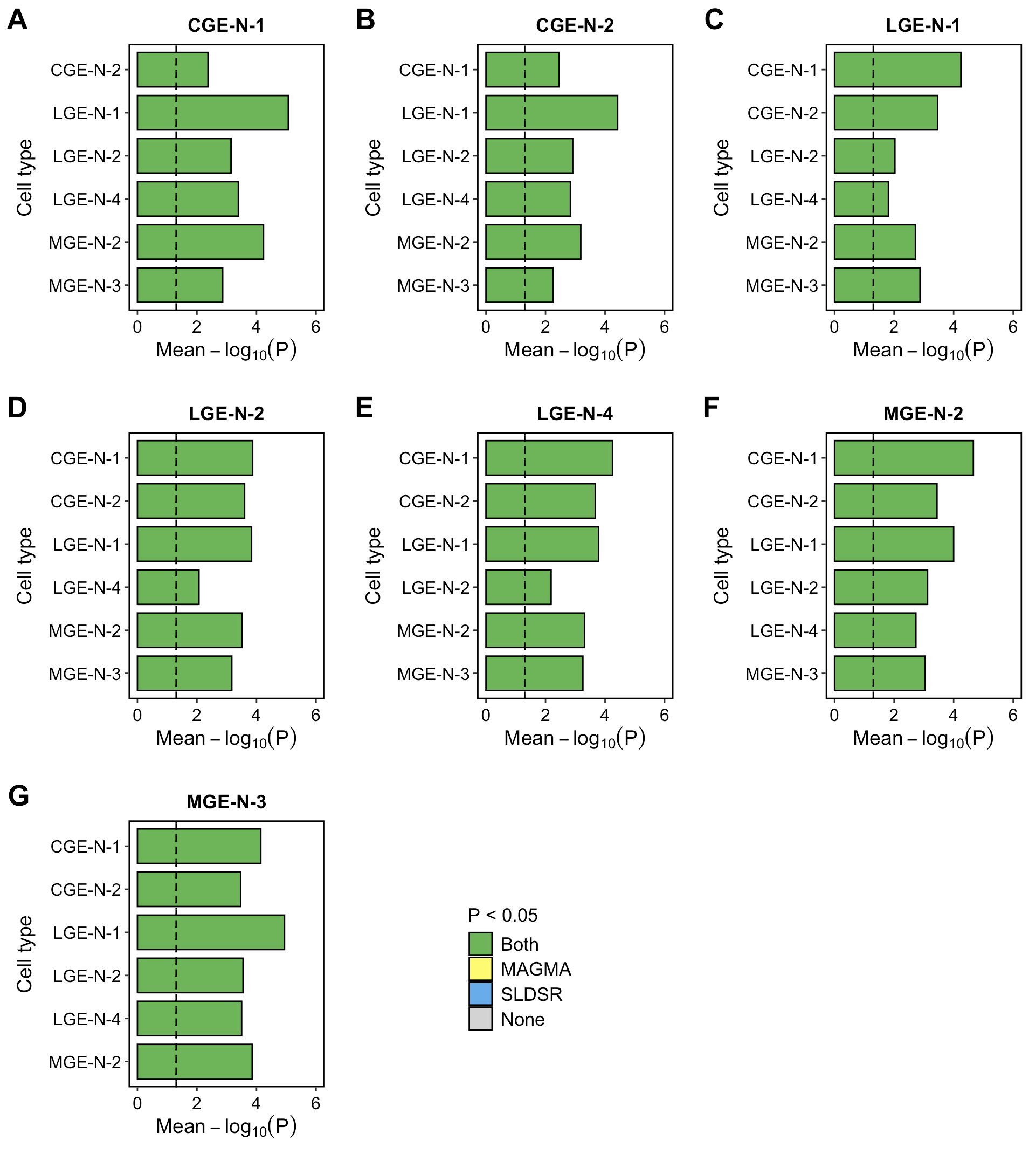


**Figure S12.** **Mean of the MAGMA (de Leeuw et al, 2015) and SLDSR (Finucane et al, 2018) -log₁₀ *P*-values for enrichment of schizophrenia associations in genes in the top expression specificity decile of each significantly enriched level 2 cell population of the ganglionic eminences, conditioning separately on genes in the top decile of each of the other 6 significantly enriched populations.** Panels A – G show the 7 tested cell populations; the Y-axes indicate the 6 other cell types on which the associations are conditioned. The dashed vertical line indicates nominal (*P* < 0.05) significance. Bars are color-coded according to whether the enrichments are significant (*P* < 0.05) for both MAGMA and SLDSR (green), MAGMA only (yellow), SLDSR only (blue) or neither test (grey). CGE-N = developing neurons from the CGE; LGE-N = developing neurons from the LGE; MGE-N = developing neurons from the MGE.

**References:**

de Leeuw CA, Mooij JM, Heskes T, Posthuma D. MAGMA: generalized gene-set analysis of GWAS data. *PLoS Comput Biol* 2015;11:e1004219.

Finucane HK, Reshef YA, Anttila V, et al. Heritability enrichment of specifically expressed genes identifies disease-relevant tissues and cell types. *Nat Genet* 2018;50:621-629


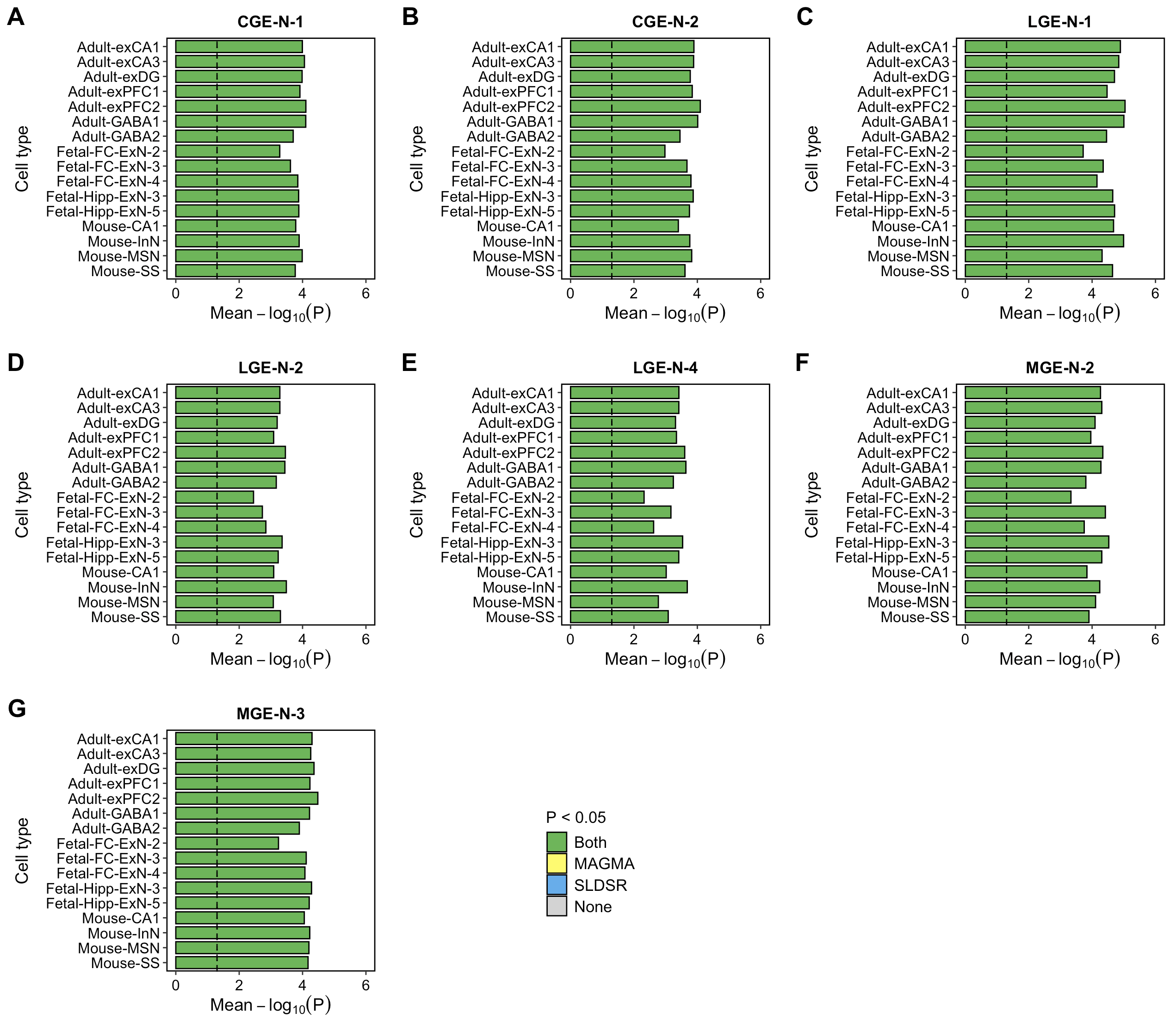


**Figure S13.** **Mean of the MAGMA and SLDSR -log₁₀ *P*-values for enrichment of schizophrenia associations in genes in the top expression specificity decile of each implicated level 2 cell population of the ganglionic eminences, separately conditioning on genes in the top specificity decile of developing glutamatergic neurons of the fetal brain (Cameron et al, 2023) and both glutamatergic and GABAergic neurons of the adult brain (Trubetskoy et al, 2022) that have also been implicated in schizophrenia through the same methods.** The dashed vertical line indicates nominal (*P* < 0.05) significance. Bars are color-coded according to whether the enrichments are significant (*P* < 0.05) for both MAGMA and SLDSR (green), MAGMA only (yellow), SLDSR only (blue) or neither test (grey). CGE-N = developing neurons from the CGE; LGE-N = developing neurons from the LGE; MGE-N = developing neurons from the MGE; ex = excitatory neuron; CA1 / CA3 = cornu ammonis areas of the adult hippocampus; DG = dentate gyrus; PFC = adult prefrontal cortex; FC = fetal frontal cortex; Hipp = fetal hippocampus; ExN = fetal excitatory neuron; InN = adult mouse inhibitory neuron; MSN = adult mouse medium spiny neuron; SS = adult mouse pyramidal neurons from the somatosensory cortex.

**References:**

Cameron D, Mi D, Vinh NN, et al. Single-Nuclei RNA Sequencing of 5 Regions of the Human Prenatal Brain Implicates Developing Neuron Populations in Genetic Risk for Schizophrenia. *Biol Psychiatry* 2023;93:157-166.

Trubetskoy V, Pardiñas AF, Qi T, et al. Mapping genomic loci implicates genes and synaptic biology in schizophrenia. *Nature* 2022;604:502-508.


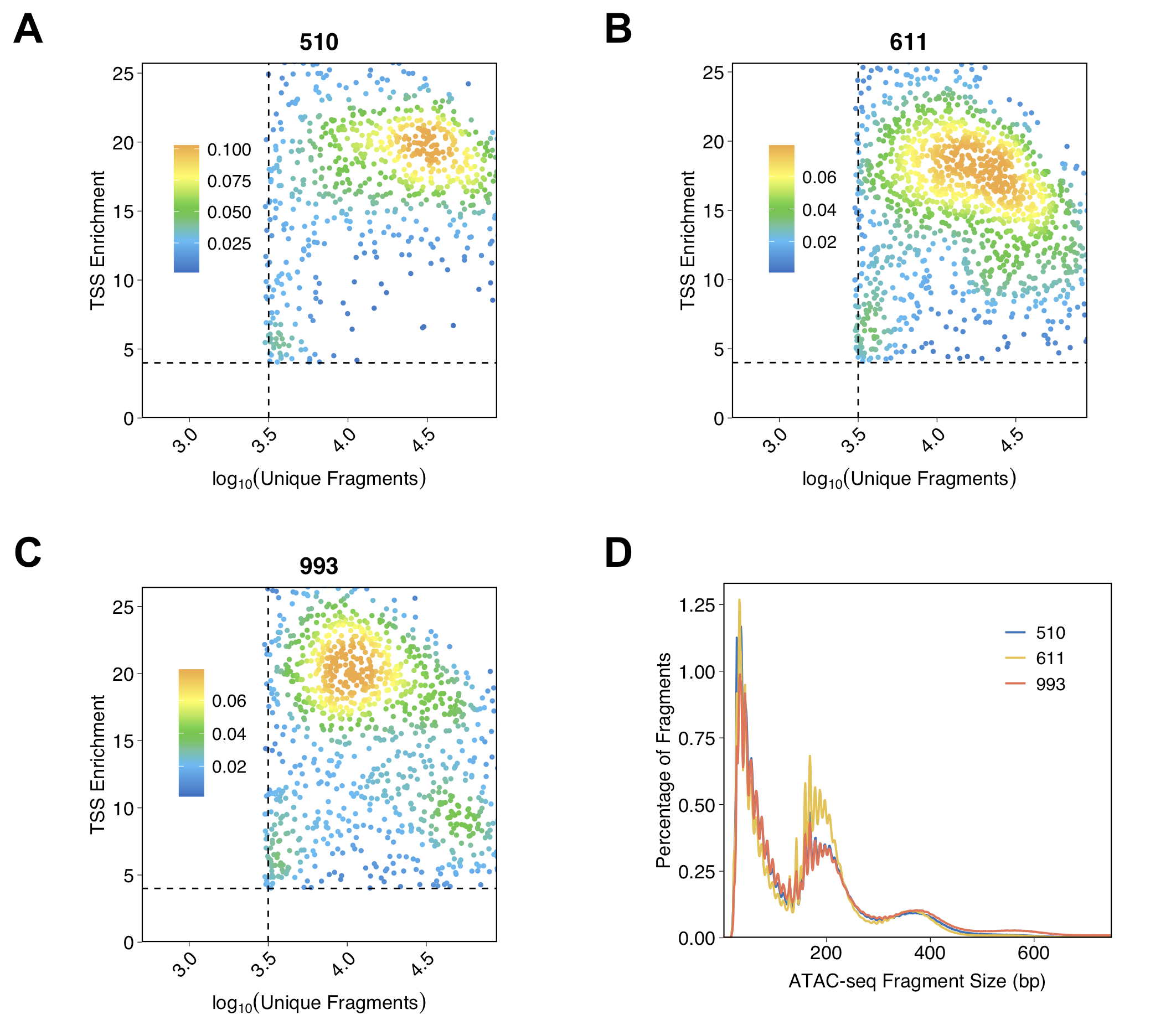


**Figure S14: Per sample quality control plots for snATAC-Seq data.** A-C) Transcription start site (TSS) enrichment and number of unique DNA fragments for snATAC-Seq data from nuclei retained from samples 510, 611 and 993. D) snATAC-Seq DNA fragment size from retained nuclei for each sample.

Analyses performed using ArchR (Granja et al, 2021).

**Reference:**

Granja JM, Corces MR, Pierce SE, et al. ArchR is a scalable software package for integrative single-cell chromatin accessibility analysis. *Nat Genet* 2021;53:403-411.


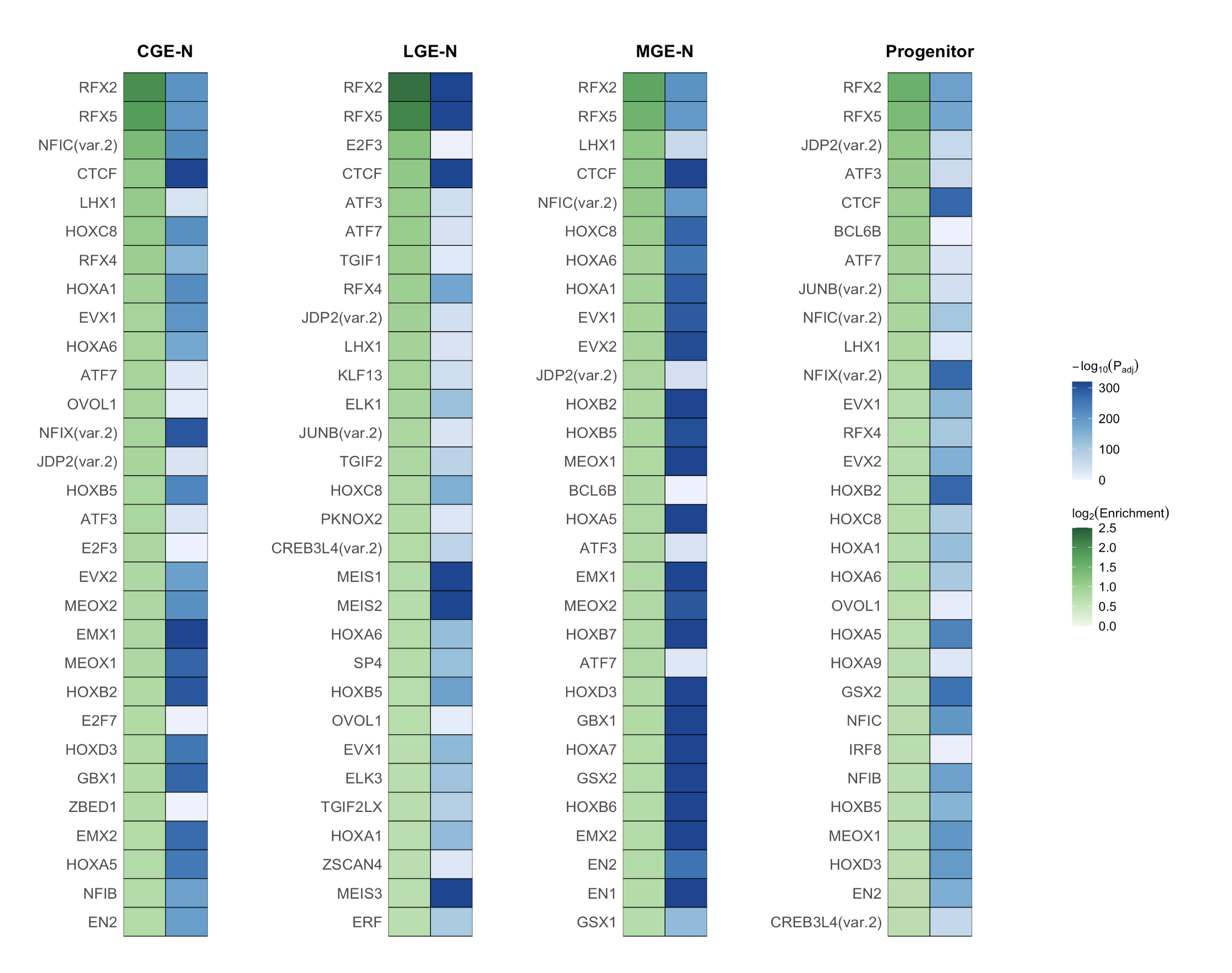


**Figure S15: The 30 most strongly enriched transcription factor binding motifs in open chromatin regions identified in each broad cell population of the ganglionic eminences.** Motifs are ordered by enrichment compared to randomly sampled, similarly sized sets of genomic sequences, accounting for differences in GC content and k-mer composition, using the R package monaLisa (Machlab et al, 2022). CGE-N = developing neurons from the CGE; LGE-N = developing neurons from the LGE; MGE-N = developing neurons from the MGE.

**Reference:**

Machlab D, Burger L, Soneson C, Rijli FM, Schübeler D, Stadler MB. monaLisa: an R/Bioconductor package for identifying regulatory motifs. *Bioinformatics* 2022;38:2624-2625.


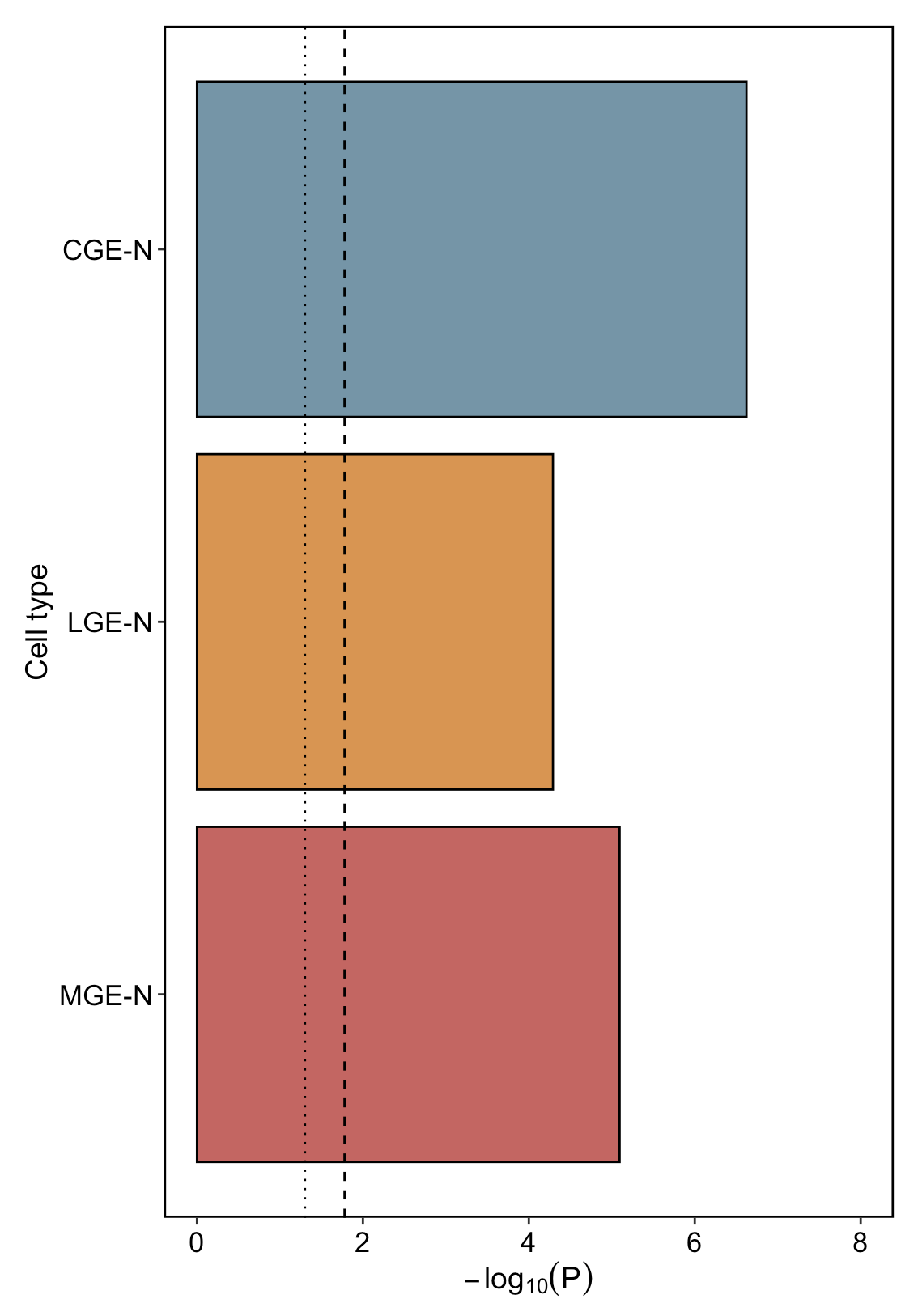


**Figure S16. -Log_10_ *P*-values for enrichment of schizophrenia SNP heritability in OCRs mapped within individual cell populations of the ganglionic eminences that had not been previously identified in bulk GE tissue (Markenscoff-Papadimitriou et al, 2020)**. Analyses were performed using SLDSR (Finucane et al, 2015); enrichment *P-*values were derived from Z-scores accounting for 53 (v1.2) baseline genomic annotations and OCRs identified in all other cell populations of the GE. The dotted vertical line indicates nominal (*P* < 0.05) significance and the dashed vertical line indicates the Bonferroni-corrected *P*-value threshold for 4 tested cell populations (*P* < 0.0125). CGE-N = developing neurons of the CGE; LGE-N = developing neurons of the LGE; MGE-N = developing neurons of the MGE.

**References:**

Markenscoff-Papadimitriou E, Whalen S, Przytycki P, et al. A Chromatin Accessibility Atlas of the Developing Human Telencephalon. *Cell* 2020;182:754-769.e18.

Finucane HK, Bulik-Sullivan B, Gusev A, et al. Partitioning heritability by functional annotation using genome-wide association summary statistics. *Nat Genet* 2015;47:1228-1235


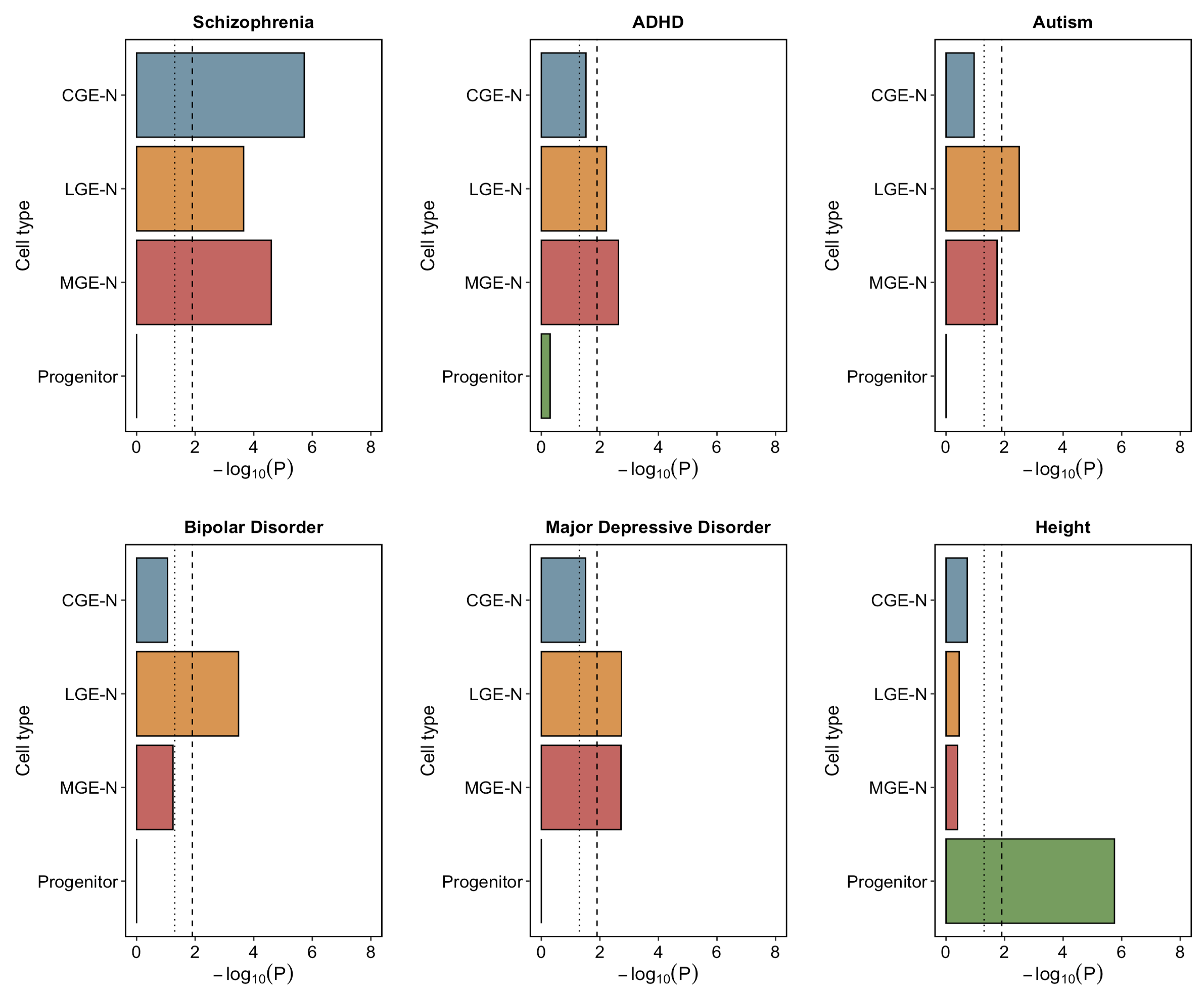


**Figure S17: -Log_10_ *P*-values for enrichment of SNP heritability for schizophrenia and comparison phenotypes in open chromatin regions mapped within individual cell populations of the ganglionic eminences**. Analyses were performed using SLDSR (Finucane et al, 2015); enrichment *P-*values were derived from Z-scores accounting for 53 (v1.2) baseline genomic annotations and OCRs identified in all other cell populations of the GE. The dotted vertical line indicates nominal (*P* < 0.05) significance and the dashed vertical line indicates the Bonferroni-corrected *P*-value threshold for 4 tested cell populations (*P* < 0.0125). CGE-N = developing neurons of the CGE; LGE-N = developing neurons of the LGE; MGE-N = developing neurons of the MGE.

**Reference:**

Finucane HK, Bulik-Sullivan B, Gusev A, et al. Partitioning heritability by functional annotation using genome-wide association summary statistics. *Nat Genet* 2015;47:1228-1235


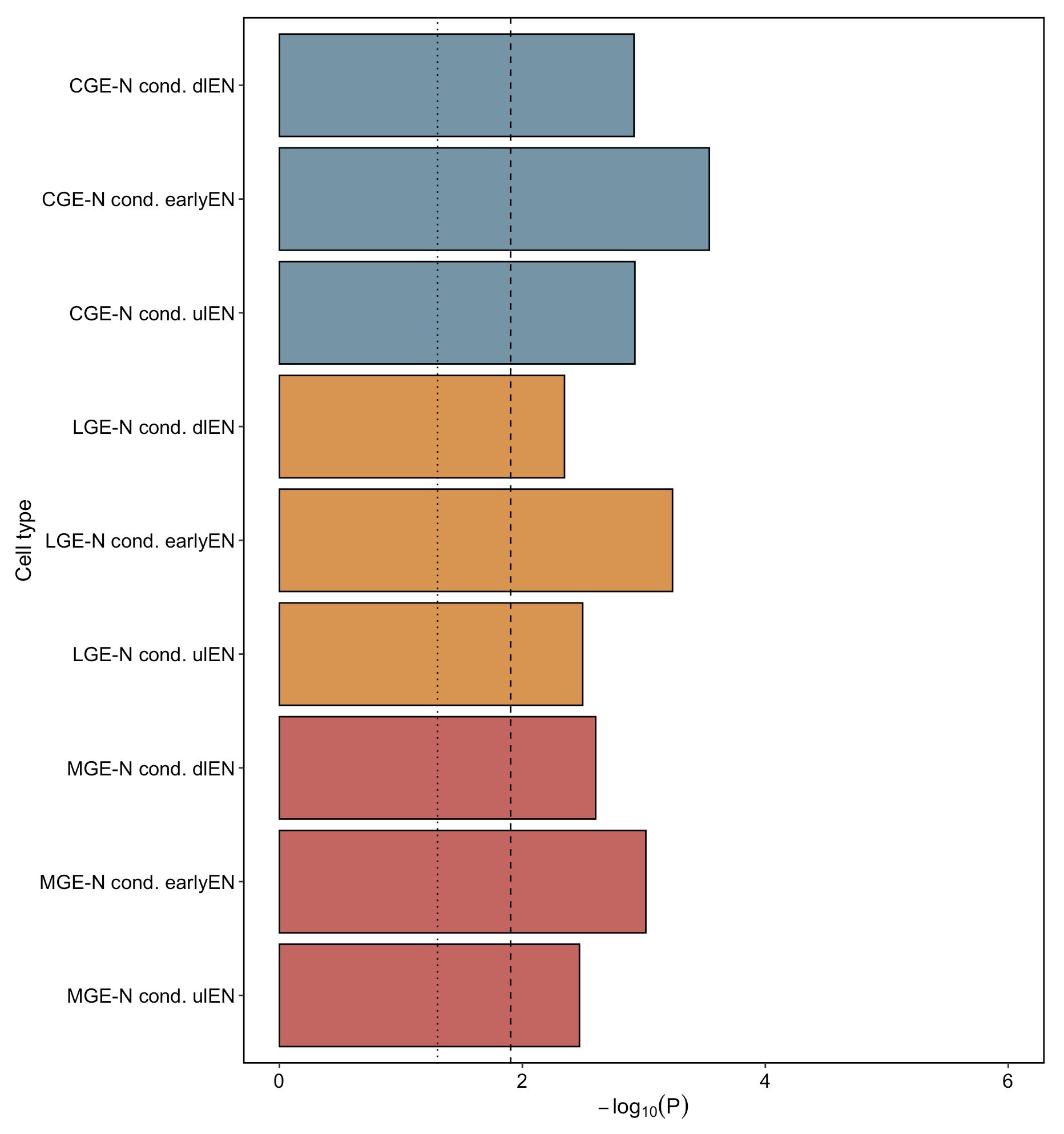


**Figure S18. -Log_10_ *P*-values for enrichment of schizophrenia SNP heritability in open chromatin regions of the ganglionic eminences, accounting for open chromatin regions identified in implicated excitatory neuron populations of the fetal brain (Ziffra et al, 2021), using SLDSR (Finucane et al, 2015)**. The dotted vertical line indicates nominal (*P* < 0.05) significance and the dashed vertical line indicates the Bonferroni-corrected *P*-value threshold for the 4 GE cell populations originally tested (*P* < 0.0125). CGE-N = caudal ganglionic eminence developing neurons; LGE-N = lateral ganglionic eminence developing neurons; MGE-N = medial ganglionic eminence developing neurons; dlEN = deep layer (cortical layers V–VI) excitatory neurons; earlyEN = early excitatory neurons; ulENs = upper layer (cortical layers II–IV) excitatory neurons.

**References:**

Ziffra RS, Kim CN, Ross JM, et al. Single-cell epigenomics reveals mechanisms of human cortical development. *Nature* 2021;598:205-213.

Finucane HK, Bulik-Sullivan B, Gusev A, et al. Partitioning heritability by functional annotation using genome-wide association summary statistics. *Nat Genet* 2015;47:1228-1235
